# Time-resolved imaging of magnetic vortex dynamics using holography with extended reference autocorrelation by linear differential operator

**DOI:** 10.1038/srep36307

**Published:** 2016-10-31

**Authors:** N. Bukin, C. McKeever, E. Burgos-Parra, P. S. Keatley, R. J. Hicken, F. Y. Ogrin, G. Beutier, M. Dupraz, H. Popescu, N. Jaouen, F. Yakhou-Harris, S. A. Cavill, G. van der Laan

**Affiliations:** 1 School of Physics and Astronomy, University of Exeter, Exeter, EX4 4QL, United Kingdom; 2 CNRS, SIMAP, F-38000 Grenoble, France; 3 Paul Scherrer Institute, 5232 Villigen, Switzerland; 4 SOLEIL Synchrotron, 91192 Saint-Aubin, France; 5 European Synchrotron Radiation Facility, F-38043 Grenoble Cedex 9, France; 6 Department of Physics, University of York, York, YO10 5DD, United Kingdom; 7 Diamond Light Source, Harwell Science and innovation Campus, Didcot, Oxfordshire, OX11 0DE, United Kingdom

## Abstract

The magnetisation dynamics of the vortex core and Landau pattern of magnetic thin-film elements has been studied using holography with extended reference autocorrelation by linear differential operator (HERALDO). Here we present the first time-resolved x-ray measurements using this technique and investigate the structure and dynamics of the domain walls after excitation with nanosecond pulsed magnetic fields. It is shown that the average magnetisation of the domain walls has a perpendicular component that can change dynamically depending on the parameters of the pulsed excitation. In particular, we demonstrate the formation of wave bullet-like excitations, which are generated in the domain walls and can propagate inside them during the cyclic motion of the vortex core. Based on numerical simulations we also show that, besides the core, there are four singularities formed at the corners of the pattern. The polarisation of these singularities has a direct relation to the vortex core, and can be switched dynamically by the wave bullets excited with a magnetic pulse of specific parameters. The subsequent dynamics of the Landau pattern is dependent on the particular configuration of the polarisations of the core and the singularities.

In recent years, vortex closure domains in magnetic thin-film elements have become the focus of great attention due to their intriguing dynamic properties. As well as being fascinating model objects – in many aspects the vortices behave like classical harmonic oscillators – they are considered as promising new magneto-electronic components, which could provide a wide range of functionalities, stretching from static non-volatile high-density data storage^1^ to dynamic skyrmion excitations^2^,^3^,^4^ and magnetic vortex generators^5^,^6^,^7^. The latter have particularly attracted much interest after it was demonstrated that, similar to spin-torque oscillators (STOs), a magnetic vortex can be driven by either AC or DC currents and perform as a microwave oscillator^7^,^8^,^9^,^10^,^11^,^12^,^13^,^14^,^15^. In comparison to an STO device, a magnetic vortex is a natural and more coherent resonating system, which is less affected by imperfections in the geometry of the material and its intrinsic parameters. However, the understanding of vortex dynamics remains complex, especially in matters regarding nonlinear effects. In case of thin film patterned magnetic elements, such as microscopic permalloy (Py, Ni_81_Fe_19_) squares or dots, the focus of research has been covering a number of topics including the understanding of the eigen-modes of gyration in single elements^1^,^11^,^12^,^15^,^16^,^17^, coupled oscillations in assemblies or patterns of elements^18^,^19^,^20^,^21^,^22^,^23^,^24^, non-linear effects^24^,^25^,^26^,^27^, manipulation of core dynamics^11^,^12^,^13^,^15^ and their practical application^7^,^8^,^12^,^13^. In many cases the investigations have been based on time-resolved imaging using optical^28^,^29^,^30^,^31^ or x-ray techniques^17^,^32^,^33^,^34^,^35^. The spatial resolution in the best case is limited to the wavelength of the optical probe convoluted with the specifics of the particular techniques and their limitations. For example, in the case of soft x-ray imaging the typical values are in the region of 20–50 nm. This is insufficient to clearly resolve the vortex core or domain walls with dimensions (~10–15 nm). In the majority of investigations these limitations were compensated by simulation studies using micromagnetic numerical tools, such as Mumax^36^, from which one can further assess the effects that may not be clearly resolved in spatial imaging. This also facilitated study of the finer structure of the vortex closures and its dynamics^11^,^26^,^37^,^38^ and to investigate non-linear effects and spin-wave phenomena associated with both the excitation and the steady states of the vortex gyration. The latter are of particular importance in technological applications, where vortex core manipulation underpins their operation (e.g. polarisation switching). Numerical work is also invaluable to understand the 3D structures of the domain closures, as these are normally ‘hidden’ for the experimental probes (e.g., in x-ray transmission experiments), because of averaging or insensitivity to in-depth magnetisation.

Here we report the first demonstration of time-resolved imaging of vortex gyration using *holography with extended reference autocorrelation by linear differential operator* (HERALDO). This relatively new technique^39^ has recently been adopted for imaging of magnetic materials^40^,^41^,^42^,^43^. Similar to most synchrotron-based magnetic probes, it uses x-ray magnetic circular dichroism (XMCD)^44^,^45^ to probe the magnetisation with element-specificity, for which magnetic contrast is observed for magnetic moments aligned parallel or anti-parallel to the x-ray wave vector. Based on the principles of holography, HERALDO uses the interference between the reference beam and the scattered light from the sample to reconstruct the real-space magnetic contrast. Compared to standard Fourier transform holography (FTH)^46^,^47^,^48^, in HERALDO the reference beam is produced by a slit rather than a hole (see Fig. 1). One of the advantages of this modification is the ability to image both out-of-plane and in-plane magnetic components by performing measurements at normal (90 degrees) and acute (e.g. 45 degrees) angles of x-ray incidence with respect to the sample respectively. Here we employ HERALDO for time-resolved imaging of the magnetic vortex gyration in thin-film magnetic Py squares. Similar to circular elements, in these structures the vortex is formed as a result of minimisation of the magnetostatic energy, forcing the magnetic moments to stay preferably in-plane of the element. However, due to the influence of the straight edges, as well as creating the vortex core, the square elements tend to form 90° domain walls, breaking the sample into four equi-sized triangular domains, forming a so-called Landau Flux Closure pattern^29^,^49^,^50^. The dynamics of the core is in this case also affected by the mobility of the domain walls and the associated magnetostatic energy. Furthermore, it was shown^51^ that the structure of the core itself and its dynamics can be greatly affected by the film thickness of the elements. Here we present a study on relatively thick (80 nm) Py elements. As well as the gyration of the core studied by x-ray imaging and micromagnetic simulations, we also examine the overall 3D structure of the magnetic configuration and its dynamics during the first cycles of excitation. In particular we focus on the associated spinwave phenomena and investigate the wave propagations localised within the domain walls of the element.

## Results

The experiments were carried out in the stroboscopic regime^12^,^16^,^17^. The overall schematics of the pump-probe measurements using HERALDO is illustrated in Fig. 2, and described in more detail in Methods. Synchronisation between the pump and x-ray probe pulses was achieved by using the master oscillator clock of the synchrotron, which served as the trigger signal for the pulse generator. To obtain images for different gyration phase, the pulses from the generator were delayed with intervals of fixed length corresponding to different time instances from the moment of initial rise (*t*_0_) of the pulse to the time of fully damped gyration (>*t*_0 _+ 30 ns). Given the limited available beamtime and the substantial acquisition time (a typical hologram took 30–40 min) a particular focus was given to the initial stage of vortex actuation and the first two cycles of gyration. Overall, we imaged 29 points starting from *t*_0_ and ending at *t*_0 _+ 10 ns, with a typical step size of 250 ps and an average spatial resolution of 30 nm (40 nm) in vertical (horizontal) direction.

**Figure 1:**
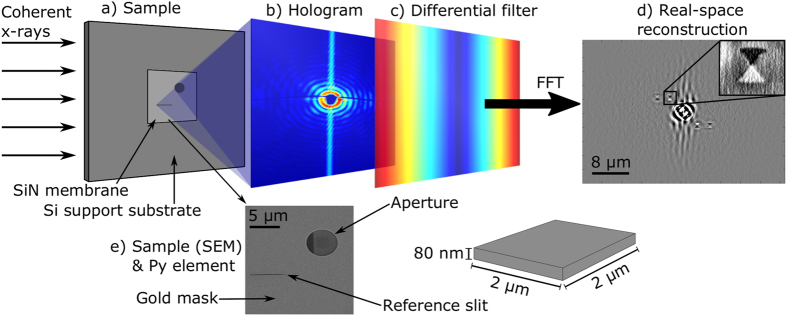
Schematics of the HERALDO setup and sample structure. (**a**) The sample on a SiN membrane. The incident x-rays diffract at the aperture hole and the reference slit. (**b**) The interference between these two beams yields a hologram that is recorded on a CCD camera. (**c**) The intensity map of the differential filter, applied along the directional derivative of the x-rays from the reference slit. (**d**) The reconstructed image after fast Fourier transform (FFT) and polarisation analysis. Inset shows a close-up of the aperture with the magnetic contrast of the Py element. (**e**) Scanning electron microscope (SEM) image from the back side of the sample showing the aperture in the 600 nm gold layer together with the reference slit. The CPW with the square Py element can be seen in the aperture through the 200 nm SiN membrane on the front of the sample.

For imaging in perpendicular orientation (see Fig. 3a) an enhanced spatial resolution of 20 nm in both vertical and horizontal directions was achieved by moving the CCD camera closer to the sample. The reconstruction of the magnetic contrast was performed in the same way as previously reported^40^,^41^. The difference between the diffraction patterns for the two polarisations of opposite circularly polarised light was multiplied by the intensity matrix representing the differential filter. The resulting image was then Fourier transformed to obtain the real-space magnetic contrast (see Fig. 1). Figure 3 shows the results of the reconstructions from the data obtained at different delay times between *t*_0_ and the x-ray probe pulse. Imaging was carried out at two different x-ray angles of incidence (90° and 45°) with respect to the sample surface, and for two different pulse amplitudes (7.7 mT and 10 mT), respectively. Figure 3a,b depict typical images of the magnetic contrast in the area of the vortex core acquired at 90° and 45°. The overlaid lines trace the positions of the core at different delay times. Figure 3c shows the vertical displacement of the core as a function of delay time, as well as the results of micromagnetic simulations, modelled using the same geometric and material parameters. Figure 3d shows the spatial positions of the core extracted from all frames imaged at perpendicular (90°) orientation of the x-rays with respect to the sample. Supplementary movies S1, S2, and S3 show the combined time-resolved images for both sample orientations.

Figure 3c shows that, as time progresses from *t*_0_, the core of the vortex is first vertically displaced (along the CPW) after which it follows a continuous precession around a new equilibrium position. This new position is determined by the amplitude of the magnetic pulse and, once the gyration is fully damped (after ~30 ns), it has the same vertical displacement as would have been induced by a constant applied field with the same magnitude as that of the pulsed field. As in experiments on circular elements (e.g.,^20^,^27^), sufficient bandwidth (Δ*f* ≈ 1/Δ*t*) for excitation of eigen-modes of the vortex gyration was provided by a pulse with a rise time of Δ*t* ≈ 1 ns. To match the amplitude of gyration with that produced in the simulation we used a Gaussian smoothing of the pulse, which leads to a further reduction of the dynamic bandwidth, but more realistically describes the experimental capabilities of the generator and the transmission lines. In the simulation, we also assumed a non-uniform structure of the field, which was inversely proportional to the distance *r* from the antenna according to the relation *B *= *B*_0_*r*_0_/(*r*_0 _+ *r*), where *B*_0_ = 7.7 mT is the field at the surface of the antenna, and *r*_0_ = 40 nm is the half-thickness of the antenna. As shown below, this non-uniform nature of the field plays an important role in the dynamics of the domain walls. Based on previous studies, the gyration frequency is mainly determined by the geometric parameters of the element and is proportional to the thickness to length aspect ratio of the square^51^,^52^. In our measurements on a square of 2 μm long and 80 nm thick, the period of the first cycle of gyration was found to be ~4.5 ns. This value is in good agreement with that obtained by the micromagnetic simulations. The reduction in the amplitude of gyration is also in agreement with the simulation, in which the damping coefficient of Py is set to its standard value of α = 0.008.

### Core structure

As mentioned above, to obtain the in-plane magnetisation contrast, which is predominant in this case, the imaging is performed at 45°, providing the required non-zero in-plane projection of the x-ray wave vector. However, regions with out-of-plane components will also result in a non-zero magnetic contrast, thereby providing more information about the overall domain structure. In particular this is important for thicker elements, in which the increased aspect ratio leads to non-linear regions with large variations of the magnetisation vector. Figure 4 shows the magnetic contrast in the vicinity of the vortex core obtained experimentally, and extracted from micromagnetic simulation. Two particular aspects arising from off-normal imaging are related to the angle of observation. Firstly, the maximum (zero) magnetic intensity is found for moments forming 45° (135°) with the surface (Fig. 4a). These are the moments that are at opposite sides of the core centre and either parallel or at 90° to the x-ray beam. Theoretically, the extension between the maximum intensity and its zero can be taken as the average dimension of the core.

Secondly, imaging at 45° results in averaging of the magnetic contrast along the lines inclined at this angle. For thicker elements this means that a projection of the vertical structure will also be present in the image. Figure 4b shows the simulation result of the experimental data in Fig. 4c, in which the averaging is taken at 45°. The area of the core shows a certain structure arising from the horizontal non-uniformity of the *M*_*x*_ component. A further examination of the simulation results for different layers of the element (Fig. 4e) reveals that the structure of the core is asymmetric towards the top and bottom layers, which are respectively mapped at their inclined projection. However, this structure is difficult to resolve, and experimentally it is exhibited only in the horizontal broadening of the core area (Fig. 4d). Examining different phases of gyration shows a similar structure, indicating that this structure is a result of the static magnetic configuration. From previous numerical studies on thick elements^53^, it is known that the structure of the core can be non-uniform, and this is generally related to the minimisation of the dipole-dipole energy across the thickness of the sample. Although this effect is relatively small, we speculate that it may play a role in the asymmetric dynamic effects of the domain walls described below.

### Domain wall dynamics

Figure 5 displays the experimental and simulated images obtained for perpendicular orientation of the x-ray beam. The contrast clearly indicates a point at the core, which was used to trace the gyration trajectory. This is particularly useful to identify the horizontal displacement, which cannot be easily extracted from the ‘in-plane’ imaging. The images are also of sufficient quality to resolve the magnetisation structure of the area around the core as well as the domain walls. Analysing the contrast of the domain walls compared to those produced in the simulations, one can see the following dynamic effects. Before the magnetic pulse is triggered, all four domain walls show a small out-of-plane magnetisation component, which has the same polarisation as the core. Once the magnetic field begins to rise after *t*_0_, two of the walls reverse to opposite polarisation. This happens through the formation of a ‘bullet-like’ excitation that propagates from the core towards the corners of the element and switches the positive component entirely or leaves a small domain close to the corners (not shown here).

The exact scenario depends on the magnitude and form of the applied magnetic pulse. A reasonably low pulse (*B*_0_ ≈ 4 mT) can switch the polarisation of both domains walls together with their corresponding corner singularities (see Figs 5 and 6). A smaller pulse amplitude can lead to switching of only one corner singularity or none at all. In the latter case all four corner singularities maintain the same polarisation, but the domain walls will partially change theirs. Once the magnetic pulse reached its maximum, the core continues along its gyration trajectory. Once the core has passed its maximum displacement, the polarisation of the domain walls typically remain the same. Generally, if the magnetic field is non-uniform, all domain walls will maintain their polarisation (two up and two down) and continue to oscillate with the core. However if the field is uniform, the bullets are still formed, but they cannot switch polarisation in the corners and after some period of sporadic motion the polarisation of all domain walls returns to its initial state (see supplementary videos S4 and S5).

It should be noted that in our simulation study the corners of the elements are ‘ideal’, so the effect of singularities is significantly pronounced. In the experiment however, due to lithographic imperfections these singularities are broadened and therefore less intense than the vortex core. This is also a likely reason why the polarisation of the corners can be switched by the onset of the pulsed magnetic field, thus leaving the combination of two black domain walls and two white domain walls that are unchanged during the core gyration (Fig. 5a). Based on the simulation results, we also note that, similar to the vortex core, the 3D structure of the domain walls is also non-uniform. Figure 6d shows how it changes across the thickness of the square. The larger contrast corresponds to the middle layers of the structure, whereas at the surfaces the effect is mostly suppressed. It was found that the formation of the bullets and their propagation strongly depends on the field gradient. If the amplitude of the field is larger at the bottom layers of the element (as is the case in our experiment), the bullets are formed in the left-hand domain walls. However, if the sign of the gradient is reversed, the bullets will be formed in the right-hand domain walls and a similar dynamic scenario is obtained, but only with opposite polarisations of the left-hand and right-hand domain walls and their singularities in the corners.

We speculate that other factors will also likely affect the demonstrated dynamics, including the initial polarisation of the core and the singularities in the corners, the vertical structure of the core, the chirality of the vortex and the temporal structure of the magnetic field pulse. Here we simulate a particular case that provides good agreement with the experimental observations. However, further combined studies of experiments and micromagnetic simulation are required to understand the phenomenon in more detail.

## Conclusions

In summary, we have demonstrated time-resolved imaging of vortex gyration using a novel technique based on x-ray holography with extended references. Using micromagnetic simulations we have confirmed the eigen frequency of the principal mode of gyration and explored the dynamics of the Landau closure domains at the onset of excitation and during the precession of the core. We showed that the 3D structure and the dynamics of the core have a direct effect on the domain walls, which can change their out-of-plane polarisation and follow the gyration of the core depending on the state of the singularities in the corners of the Landau pattern. We also showed that the change in polarisation is accomplished via the formation of ‘bullet-like’ excitations, which propagate within the domain walls and can also lead to switching the singularities at the corners. The latter are energetically related to the polarisation of the core and can reduce the energy required for switching its state. These effects are important for further understanding these systems because the manipulation of the core and its dynamics are key factors in the prospective technology utilising vortex gyration.

## Methods

### Sample preparation

**Figure 2:**
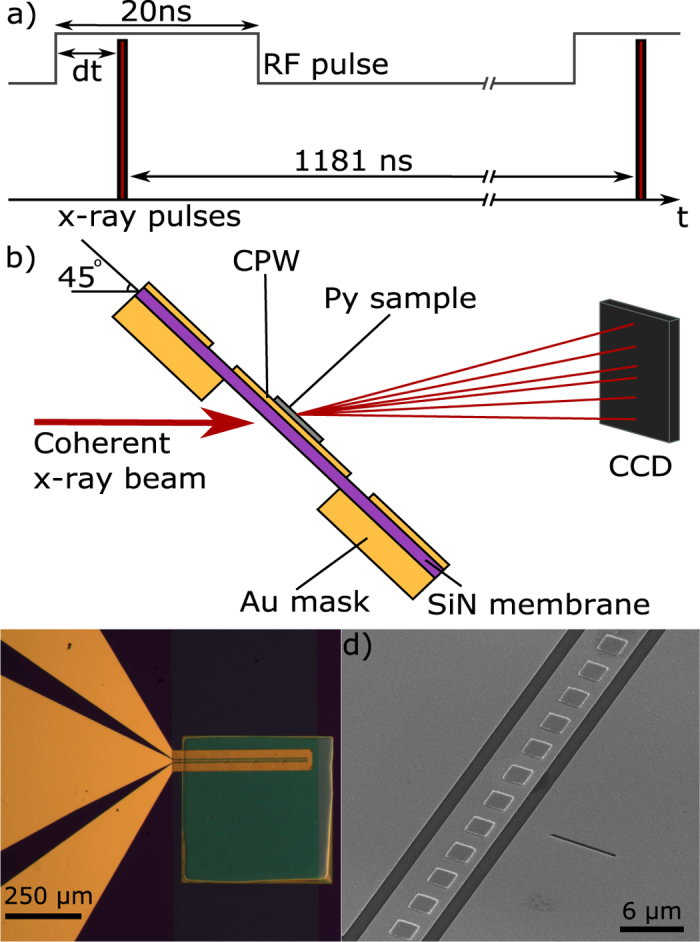
Experimental set-up and time structure of the experiments. (**a**) Pump-probe time structure used for the stroboscopic imaging. (**b**) Sample geometry with respect to the x-ray beam for 45° imaging (for perpendicular imaging the sample surface is at 90° to the x-ray beam). (**c**) Optical image of the sample showing the gold CPW on the Si substrate and SiN window. (**d**) SEM image showing the Py squares, CPW structure and reference slit. The aperture is on the rear side of the sample along the CPW core. Each CPW contains multiple apertures and reference slits separated by 100 μm.

**Figure 3:**
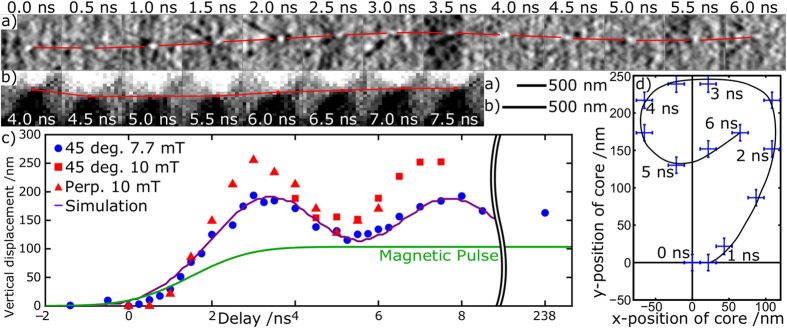
Vortex gyration. Close-up of the reconstructed magnetic contrast around the core taken at different values of the delay time d*t* for (**a**) 90° and (**b**) for 45° (3.7 V pulse) angle-of-incidence. The lines trace the vortex core position. (**c**) The vertical displacement of the vortex core at different delay times. The purple line shows vertical displacement extracted from micromagnetic simulations. The simulated magnetic pulse profile is shown by the green line. The point at 238 ns delay gives the vertical position of the core after being fully damped. The pulse length for this particular point was increased to 500 ns to allow verifying the vertical position of the core in this case. (**d**) The position of the vortex core in the *xy* plane at various delay times imaged in perpendicular orientation.

The samples were prepared on a 500 μm × 500 μm × 200 nm Si_3_N_4_ membrane, which served as substrates for lithographically produced CPWs and Py squares. In both cases, the standard electron-beam lithography (EBL), metal deposition and lift-off techniques were used to obtain free standing Py elements. A dual Focussed Ion beam (FIB) system was used to form the apertures and reference slits. The Py squares were 2 μm × 2 μm × 80 nm and had an edge-to-edge separation of 2 μm which ran along the entire length of the CPW core. A 600 nm thick gold film served as a mask and was evaporated onto the underside of the sample in order to block the x-rays in the imaging measurements. Each device had a 3.5 μm aperture milled through the gold mask with a FIB down to the silicon nitride membrane. Each CPW contained 5 devices separated 100 μm apart from each other along the CPW core (see Fig. 2(d)). Reference slits were milled throughout the mask, membrane and CPW ground strip, the centre of which was positioned 3 μm down and 7 μm to the side of the centre of the aperture. Slits were 6 μm long and on average 35 nm wide.

### HERALDO measurements

The initial tests and optimisation of x-ray holographic measurements were carried out on the beamlines ID32 (ESRF) and SEXTANTS (SOLEIL)^54^ using 16 bunch (~70 mA) and 8 bunch (~80 mA) filling modes. The final results presented here were taken at SEXTANTS, using a single bunch mode with an average current of 17 mA. (SOLEIL). In order to produce the pulsed magnetic field inside the elements we used a shorted coplanar wave guide (CPW) antenna, which was formed from a Au (80 nm) film deposited onto the SiN membrane using photolithography. The width (5 μm) and separation (1 μm) of the CPW were chosen to maintain the characteristic impedance of the microwave electronics to enhance the current density within the antenna and the resulting magnetic field. The pumping was achieved with magnetic field pulses, which were generated by a 330 MHz 3.8 V pulse pattern generator (Agilent HP 81110A + 81112A). The pulse length was 20 ns. The two pulse amplitudes (7.7 mT and 10 mT) were produced with the generator output of 3.0 V and 3.7 V respectively. To allow for a sufficient time of gyration, the rise-time of the pulses was set to the minimum possible setting of the generator (~800 ps) to provide the necessary bandwidth excitation in the MHz/GHz region^11^,^16^,^52^. The individual x-ray pulses (in single bunch mode) were separated by 1181 ns and had an average width of 25–30 ps. The transmitted x-rays were recorded on a CCD camera positioned on the optical axis behind a beam stop for the direct, undiffracted beam. The distance from the camera to the sample was set to 25 cm, resulting in a minimum spatial resolution of 20 nm. To obtain holographic images the sample had a reference slit that was isolated from the imaged area by a distance corresponding to twice the aperture diameter, and within the x-ray coherence length (~25 μm). The interference pattern of the direct and diffracted x-rays was recorded on the CCD for left- and right-circularly polarised x-rays, which were subtracted and then operated on by the directional derivatives and Fourier transformed to obtain the real-space image. The magnetic images were taken at a photon energy of 707 eV coinciding with the Fe *L*_3_ absorption edge.

### Micromagnetic Simulations

**Figure 4:**
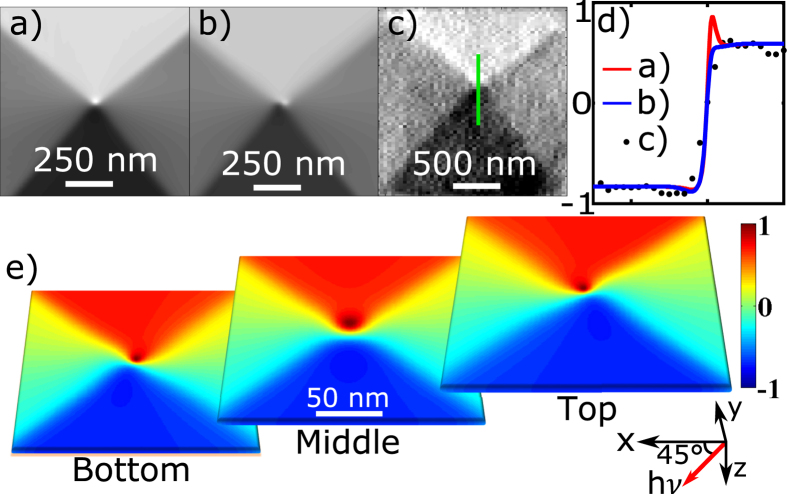
The core structure. Simulated normalised magnetic contrast with averaging out the thickness in (**a**) perpendicular orientation and (**b**) over 45° direction. (**c**) Reconstructed experimental image obtained for the same delay time. (**d**) Vertical intensity profiles through the centre of the core for all three images a, b and c. Positive values correspond to magnetisation vector parallel to the x-ray wave vector. (**e**) Simulated images of the magnetic contrast for different layers within the structure. From left to right, the images represent the bottom 1^st^, middle 8^th^ and top 16^th^ layer of a 16-cell thick simulation grid (cell size is 3.9 × 3.9 × 5 nm^3^). Colour coding: Positive (Red) and negative (Blue) normalised magnetic contrast.

**Figure 5:**
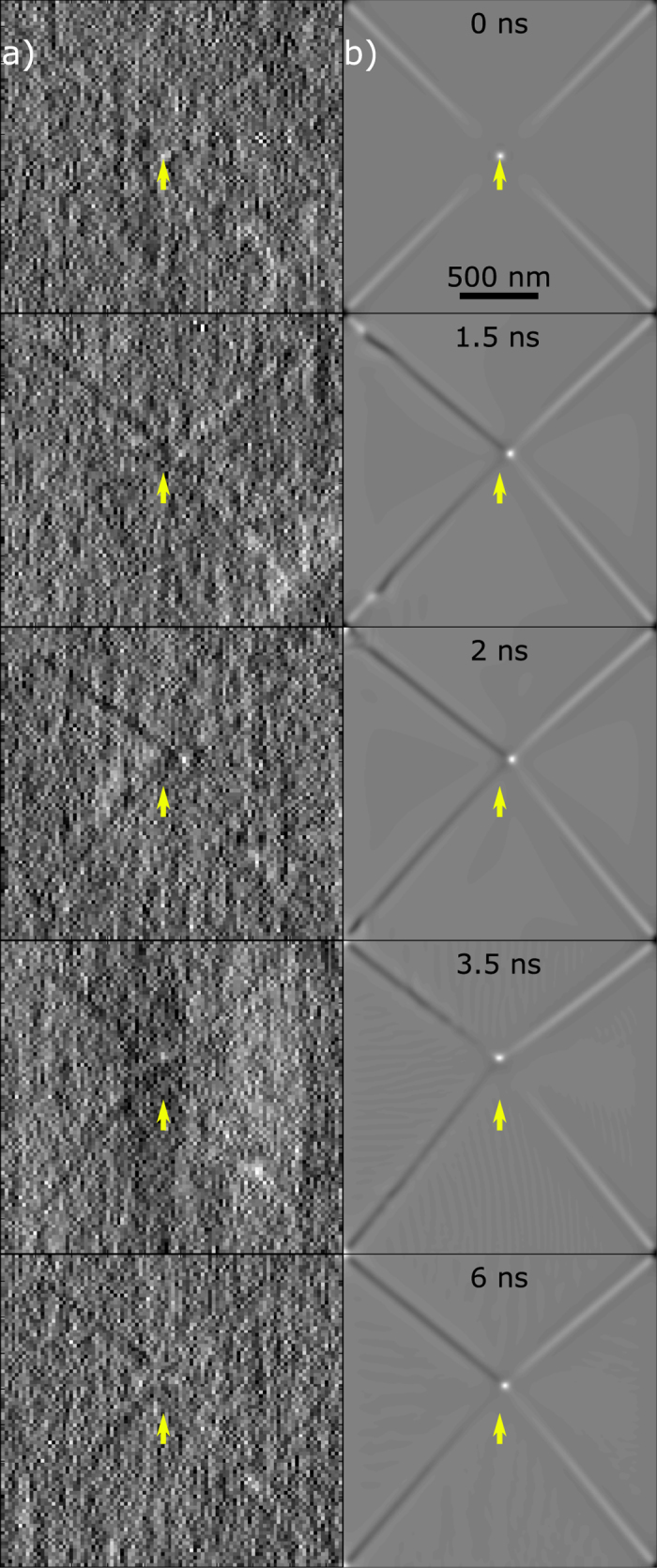
Domain wall dynamics. (**a**) Experimental and (**b**) simulated magnetic contrast of the domain structure imaged in perpendicular orientation at different delay times (from top to bottom): 0.0, 1.5, 2.0, 3.5 and 6.0 ns. The frames represent different stages of the pulse rise and gyration. The domain walls at the left-hand side in each image are predominantly ‘black’ (magnetised downwards), whereas those at the right-hand side are always ‘white’ (magnetised upwards). Yellow arrows have been inserted to point to the equilibrium position of the core.

**Figure 6:**
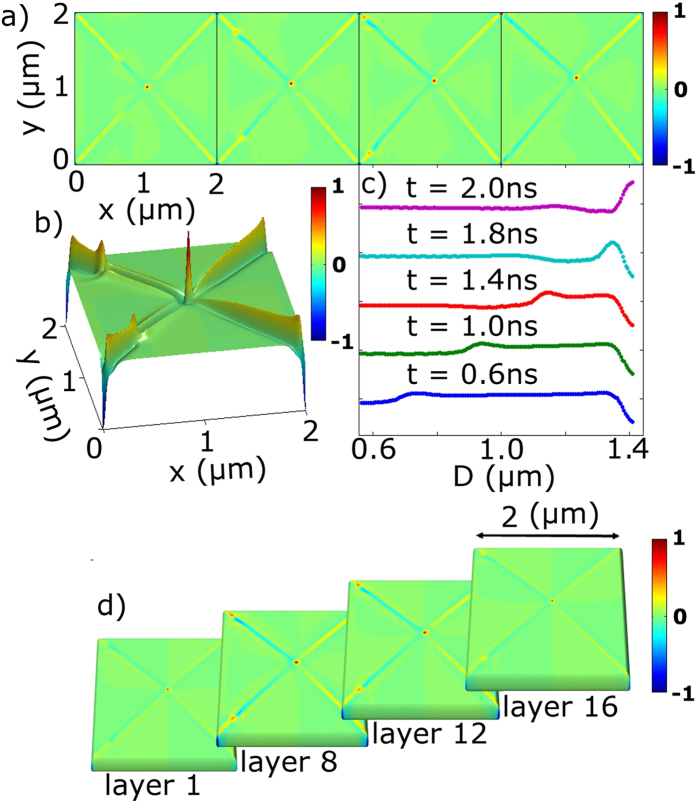
Simulated images of the domain wall structure. (**a**) Perpendicular component of the magnetisation at different delay times *t*_0_ + : 0.3, 1.3, 1.8 and 2.3 ns. (**b**) 3D depiction of the out-of-plane magnetic component at 1.4 ns delay. A localised wave ‘bullet’ is formed at the edge of the positively polarised part of the domain wall and propagates within the wall towards the corner. (**c**) Intensity scans within the domain wall for different delay times demonstrating the profile of the ‘bullet’ as it approaches the singularity at the corner. (**d**) The simulated structure of the domain walls for different layers throughout the thickness of the element. The wave ‘bullet’ structure is more pronounced in the middle layers.

Micromagnetic simulations of 2 μm permalloy squares with thickness 83 nm were performed using the MuMax micromagnetic solver^33^. Parameters for permalloy (saturation magnetisation *Ms* = 8 × 10^5 ^A m^−1^, exchange constant = 1 × 10^−11 ^J m^−3^ and Gilbert damping = 0.008) were chosen with cell-size *x *= 2000/512 nm, y = 2000/512 nm, *z* = 83/16 nm and negligible magnetocrystalline anisotropy. Vortex precession was induced by a step pulse with maximum amplitude(s) of 7.7 mT and 10 mT and rise-time ~1 ns. The field profile decays as 1/(*r*_0_ + *r*) through the thickness of the square element with *r*_0_ = 40 nm representing the half thickness of the antenna. The lateral variation of the field (close to the edges of the central CPW conductor) was neglected. The dot product of the vector magnetisation was calculated with respect to a 135 degree (see Fig. 4b) and perpendicular (see Figs 5 and 6) orientation of the x-ray and the magnetisation was averaged across the thickness at the corresponding angle.

## Additional Information

**How to cite this article**: Bukin, N. *et al*. Time-resolved imaging of magnetic vortex dynamics using holography with extended reference autocorrelation by linear differential operator. *Sci. Rep.* **6**, 36307; doi: 10.1038/srep36307 (2016).

**Publisher’s note:** Springer Nature remains neutral with regard to jurisdictional claims in published maps and institutional affiliations.

## Supplementary Material

Supplementary movies S1

Supplementary movies S2

Supplementary movies S3

Supplementary movies S4

Supplementary movies S5

Supplementary Information
