# Supplementary figures and images for: Time-resolved imaging of magnetic vortex dynamics using holography with extended reference autocorrelation by linear differential operator
Source: Sci Rep. 2016 Oct 31;6:36307. doi: 10.1038/srep36307 (PMC5087091; doi:10.1038/srep36307)

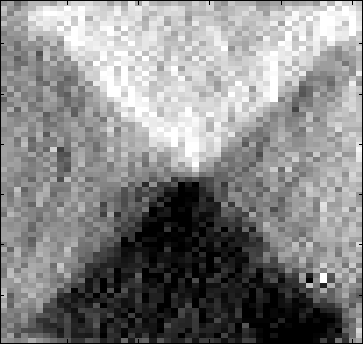

Supplement: Supplementary movies S1 [file srep36307-s1.gif]

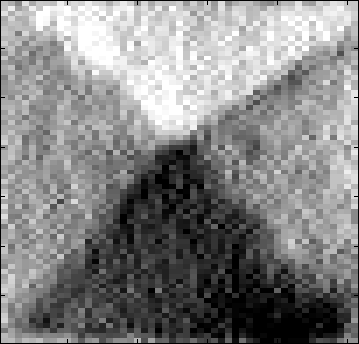

Supplement: Supplementary movies S2 [file srep36307-s2.gif]

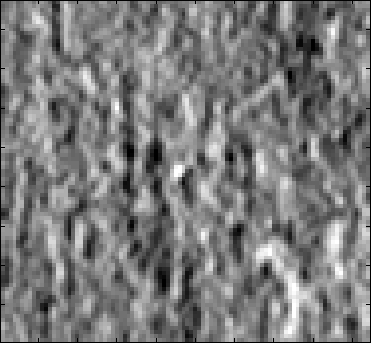

Supplement: Supplementary movies S3 [file srep36307-s3.gif]

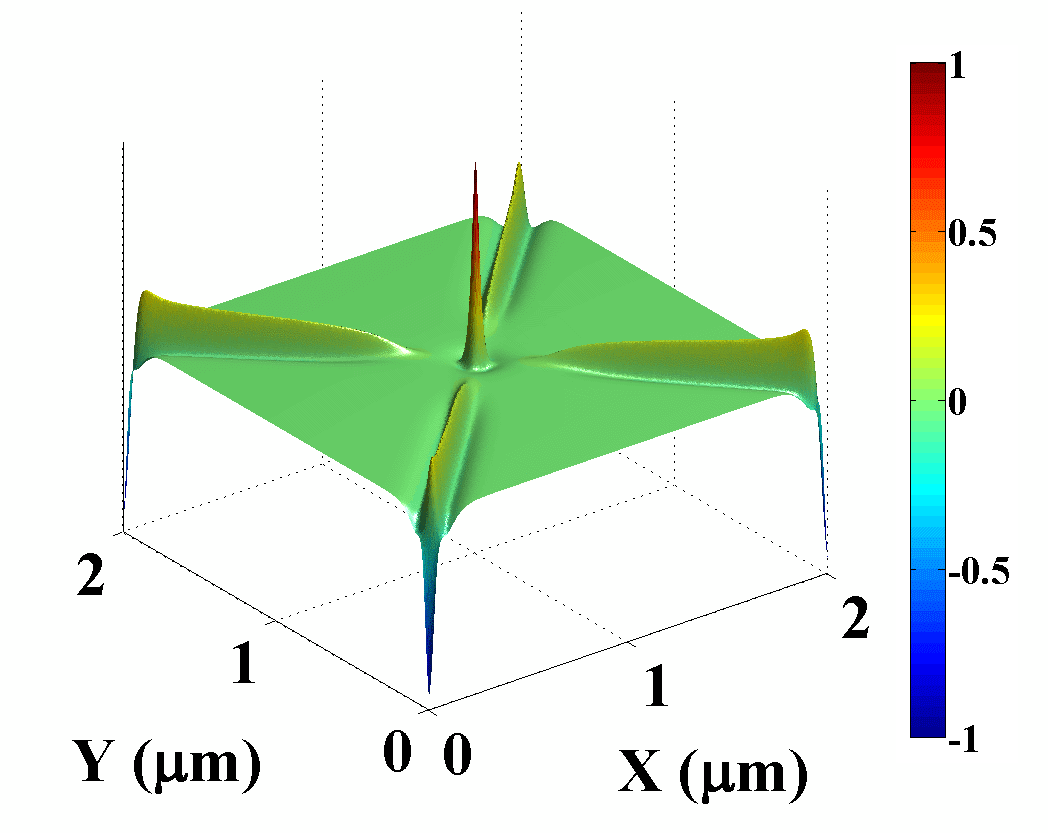

Supplement: Supplementary movies S4 [file srep36307-s4.gif]

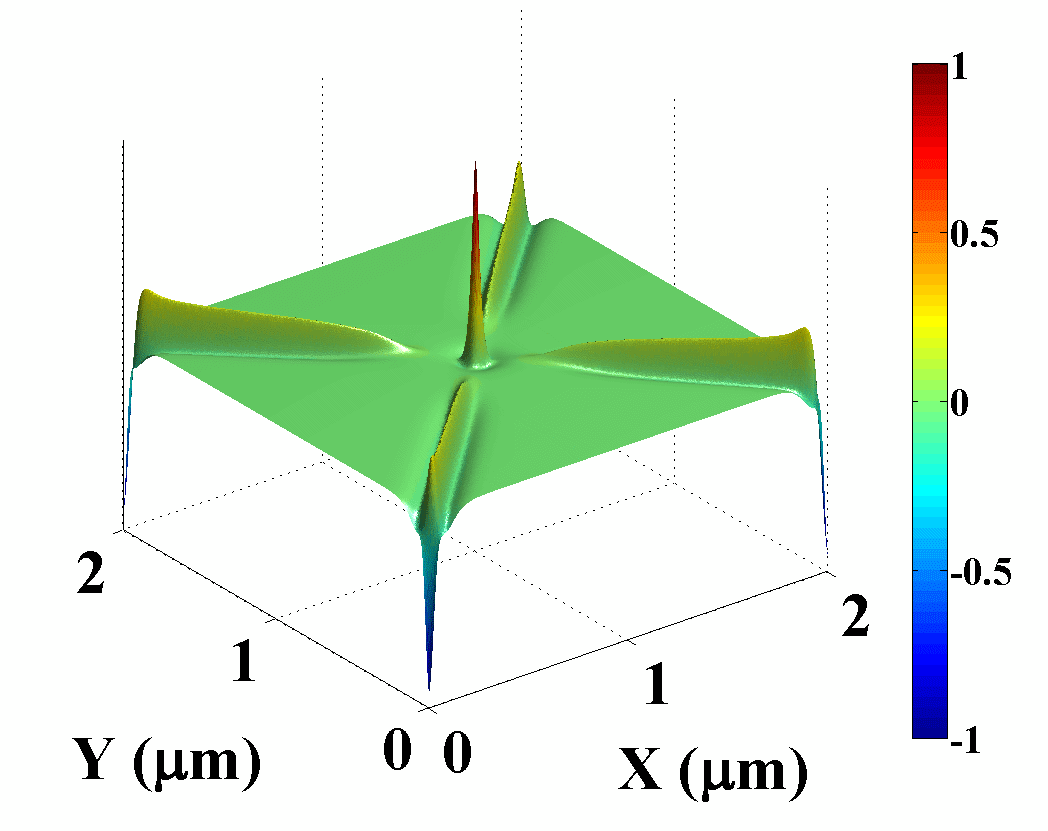

Supplement: Supplementary movies S5 [file srep36307-s5.gif]
