# Supplementary material for: Time-resolved imaging of magnetic vortex dynamics using holography with extended reference autocorrelation by linear differential operator
Source: Sci Rep. 2016 Oct 31;6:36307. doi: 10.1038/srep36307 (PMC5087091; doi:10.1038/srep36307)
Supplement: Supplementary Information [file srep36307-s6.pdf]

Time-resolved imaging of magnetic vortex dynamics using holography with extended reference autocorrelation by linear differential operator.

N. Bukin[1], C. McKeever[1], E. Burgos-Parra[1], P. S. Keatley[1], R. J. Hicken[1], F. Y. Ogrin*[1], G. Beutier[2], M. Dupraz[3], H. Popescu[4], N. Jaouen[4], F. Yakhou-Harris[5], S. A. Cavill[6] and G. van der Laan[7]

[1]School of Physics and Astronomy, University of Exeter, Exeter, EX4 4QL, United Kingdom.
[2]CNRS, SIMAP, F-38000 Grenoble, France.
[3]Paul Scherrer Institute, 5232 Villigen, Switzerland.
[4]SOLEIL Synchrotron, 91192 Saint-Aubin, France.
[5]European Synchrotron Radiation Facility, F-38043 Grenoble Cedex 9, France.
[6]Department of Physics, University of York, York, YO10 5DD, United Kingdom.
[7]Diamond Light Source, Harwell Science and innovation Campus, Didcot, Oxfordshire, OX11 0DE, United Kingdom.

*Correspondence e-mail: f.y.ogrin@exeter.ac.uk

Supplementary movies

Movie S1: Movie of all images taken for the sample at 45° to the x-ray beam starting from -1.4 ns and finishing at 8.5 ns delay from t_0. The vortex core is initially positioned in the centre of the element. At time t_0 it starts moving upwards, reaches maximum deflection and then makes two oscillations around the new stationary point. The vertical position of the core from this animation is shown in figure 3c (blue points) and corresponds to the pulse strength generated at 3 V on the RF generator.

Movie S2: Movie of 8 images taken for a larger magnetic pulse. The sample is at 45° to the x-ray beam. The zoomed-in frames are shown in figure 3b, with the corresponding vertical displacements of the core shown in figure 3c (red squares). The vortex core undergoes one cycle of gyration around the new stationary position determined by the DC component of the magnetic pulse.

Movie S3: Movie of the images for the sample perpendicular to the x-ray beam. The frames correspond to the delay times starting from 0 ns to 6 ns after t_0. Zoomed-in images of the core, together with the vertical and horizontal displacements are shown in figure 3a, 3c, and 3d respectively. The vortex core initially moves upwards and to the right and then undergoes one cycle of gyration around its new offset position. The domain walls are also visible, the two to the right of the core being white and the two to the left being black similarly to the simulations shown in figures 5 and 6 and in animation S4.

Movie S4: Simulated movie showing the initial ground state of the Landau flux-closure state and the dynamics present once an externally applied non-uniform magnetic field excites vortex gyration. The initial state has the core with a +1 polarity and the corners each containing a singularity with -1 polarity. All four domain walls have a slight perpendicular component that is polarised in the same direction as the core. Following the onset of the pulse, there is a formation of 'bullet-like' excitations in two domain walls which propagate towards the corners alongside the walls. While propagating the bullets change polarisation of the walls from positive to negative. When reaching the corners the

bullets also switch polarisation of the corners from negative to positive. The vortex core continues the gyration, maintaining the new set of polarities: core and two singularities at the corners with +1 and two singularities at the remaining corners with -1. The polarisation of domain walls remains modified: two ‘up’ (+1) and two down (-1).

Movie S5: Simulated movie showing a similar scenario to S4, but now in the case when the applied field is uniform across all of the sample. The initial stages of the simulation are similar to that in S4. Following the onset of the pulse after t_0 two bullets are formed and also propagate towards the corners, however in this case they do not change polarisation of the singularities at the corners and appear to be ‘reflected’ back towards the core. After this point the overall behaviour of the domain walls becomes more incoherent and there is no well-defined structure that can be stable enough to maintain its integrity during the full gyration cycle.
